# Utilizing the NIMHD Framework to Explore Barriers and Facilitators to Lung Cancer Screening Among Black Adults in NYC

**DOI:** 10.21203/rs.3.rs-7909142/v1

**Published:** 2026-01-16

**Authors:** Wynta Alexander, Mei Maeda, Jaime L. Gilliland, Jamie S. Ostroff, Lisa Carter-Bawa, Lina Jandorf, Victoria Frederico, Lesia M. Ruglass

**Affiliations:** The Graduate Center, CUNY; The City College of New York, CUNY; Memorial Sloan Kettering Cancer Center; Memorial Sloan Kettering Cancer Center; Hackensack Meridian Health; The Icahn School of Medicine at Mount Sinai; Memorial Sloan Kettering Cancer Center; The City College of New York, CUNY

**Keywords:** lung cancer screening, Black adults, community engagement, qualitative research

## Abstract

**Background:**

Lung cancer is the leading cause of cancer-related death in the United States, with Black individuals experiencing the highest incidence and mortality rates. Despite the benefits of early detection through low-dose computed tomography (LDCT), lung cancer screening rates remain disproportionately low among Black adults. This study explores barriers and facilitators to inform culturally tailored interventions that promote equitable screening uptake.

**Methods:**

Guided by the National Institute on Minority Health and Health Disparities (NIMHD) research framework, we conducted semi-structured interviews with screening-eligible Black adults in New York City (NYC). Participants were recruited through community canvassing and stakeholder partnerships. Transcripts were analyzed using thematic content analysis to identify key themes across individual, interpersonal, community, and societal levels.

**Results:**

Analysis of 11 interviews identified four key thematic constructs inclusive of participants’ perceptions of barriers and facilitators to lung cancer screening in the Black community. Preparation is concerned with community members’ lack of knowledge about lung cancer and screening, and strategies for community outreach and education. Partnership emphasizes the ways in which discrimination, mistrust, and stigma impact how the Black community engages in healthcare and other collaborative relationships to promote uptake of lung cancer screening. Prioritization highlights individual and community perceptions of healthcare and screening prioritization and demonstrates a need for greater prioritization of lung cancer screening in the healthcare setting. Finally, placement stresses the importance of embedding screening services within communities for improved access.

**Conclusion:**

Addressing lung cancer screening disparities requires multilevel strategies that enhance provider communication, expand access, and engage community partners. Culturally responsive approaches, such as social network engagement, mobile screening units, stigma reduction, and targeted education are essential to increasing awareness and early detection in Black communities.

## Introduction

In the United States, lung cancer is the leading cause of cancer death [1] and is the second most common cancer [2–3] in both men and women. Smoking cigarettes remains the primary risk factor [4] contributing to 80–90% of lung cancers [5–6]. Despite overall advances in prevention and early detection, persistent racial and ethnic disparities in lung cancer outcomes remain a pressing public health concern [7]. Specifically, Non-Hispanic (NH) Black or African American (hereafter referred to as Black) males have the highest rates of age-adjusted lung cancer incidence between 2004 and 2014 among all racial and ethnic groups [8]. Black individuals are also more likely to present at advanced lung cancer stage [9–10], further contributing to poor outcomes.

To detect lung tumors at earlier, more treatable stages, the U.S. Preventive Services Task Force (USPSTF) recommends low-dose computed tomography (LDCT) of the chest for high-risk individuals [11–12]. However, overall uptake remains alarmingly low, with only 16% of those at high risk receiving annual screening [13]. Among eligible Black individuals, screening rates are even lower, at only 1.7% [14–15]. Poulson et al. (2022) observed a 44% lower likelihood of undergoing screening among Black individuals compared to their White counterparts [16]. These disparities are rooted in multi-level barriers, including systemic racism, healthcare mistrust, limited access to care, and exclusion from clinical trials [17].

To enhance our understanding of the multi-level factors contributing to lung cancer screening disparities, this study applied the National Institute on Minority Health and Health Disparities (NIMHD) Research Framework [18] which emphasizes the role of structural factors, such as discrimination, socioeconomic inequality, and policy-level barriers in influencing health disparities among Black populations. The NIMHD framework offers a valuable lens through which the current study explores how individuals make sense of the complex factors surrounding lung cancer screening.

## Methods

### Study Design

This qualitative study explored multi-level influences from individual to societal across biological, behavioral, built environment, sociocultural, and healthcare system domains on lung cancer screening. We conducted semi-structured interviews with Black, screening-eligible adults to explore barriers and facilitators to lung cancer screening. The NIMHD Framework [18] guided our thematic analysis to situate participants’ experiences within the broader social and structural contexts shaping screening behavior. All study procedures were approved by the Institutional Review Boards at Memorial Sloan Kettering (protocol #X21–060) and the City University of New York (protocol #2022 − 0357).

### Participants and Recruitment

**Figure 1:**
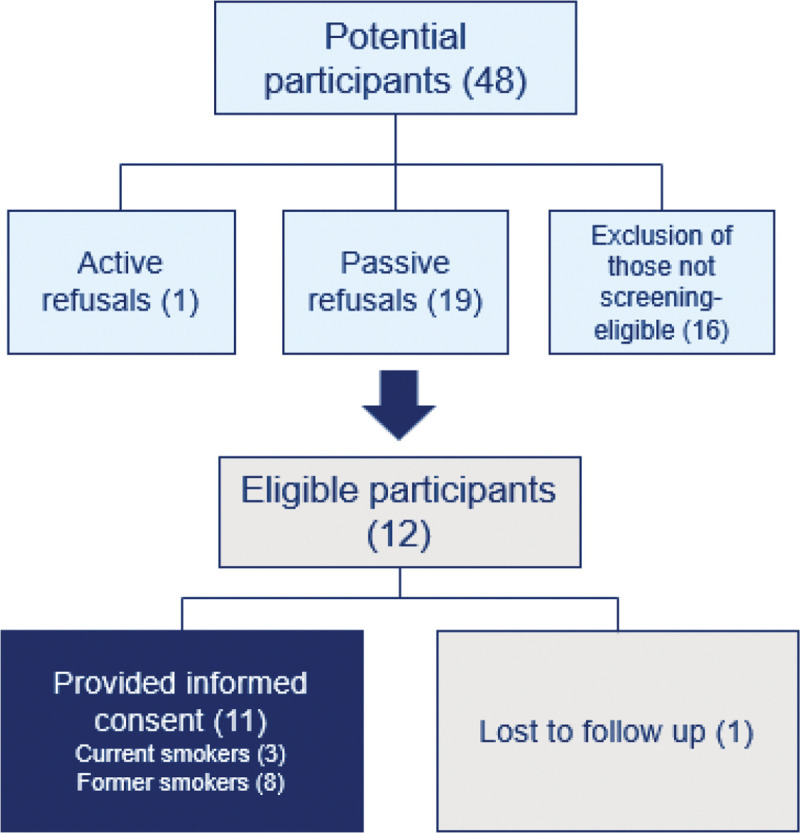
Sampling framework

The study included individuals who met eligibility criteria for lung cancer screening: aged 50–80, with a 20-pack year history or more of smoking who either currently smoke or have quit smoking within the past 15 years [19], and who self-identified as Black and resided in the NYC area. Participant characteristics can be found in Table 1. Members of the study team (WA, MM) conducted in-person community canvassing in the NYC metropolitan area for potential participants. Employing purposive and snowball sampling, eligible participants were asked to participate in an interview and to recommend others for participation. A recruitment flyer was posted on community center boards, handed out during in-person canvassing, and given to participants to share with their communities. The study team also collaborated with community stakeholders in the Harlem, NYC area. Individuals interested in the study reached out to the study team. Of 48 potential participants, 11 were consented to the study, as seen in Fig. 1.

### Qualitative Data Collection & Analysis

A semi-structured interview guide was created to explore knowledge, barriers, and facilitators of lung cancer screening within participants’ communities. Based upon previous community-engaged work conducted by the Witness Project^®^ [20] to promote lung cancer screening, open-ended questions allowed participants to share knowledge and attitudes towards lung cancer screening (e.g. “*Who is eligible for lung cancer screening?*”), community-specific factors related to lung cancer screening uptake and implementation (e.g. “*What factors are important to implementing lung cancer screening in the Black community?*”), facilitators of lung cancer screening (e.g. “*What things would help Black individuals who are eligible get screened?*”), and barriers to lung cancer screening (e.g. “*What things do you see stand in the way of eligible Black individuals getting screened?*”)

Facilitated by two trained and supervised graduate-level research fellows (WA and MM), 11 semi-structured interviews were conducted via Zoom video conferencing platform (Zoom Video Communications, Inc, San Jose, CA) lasting approximately 45 minutes between October 2022 and June 2023 and were conducted according to established methodologic guidelines [21]. Thematic saturation was achieved by conducting enough interviews until no new themes emerged during analysis [22].

Each interview was audio recorded and transcribed verbatim. All transcripts were analyzed using deductive and inductive thematic content analysis [23–25]. The coding team was led by a qualitative methods specialist (QMS) (JG) and included the graduate-level research fellows. First, the coding team individually reviewed a subset of transcripts to identify domains of interest and create codes. Once agreed upon, these codes were consolidated and used to code subsequent transcripts. The team then independently coded each transcript in NVivo 14, highlighting significant statements within each domain. This iterative process involved the coding team meeting regularly with the larger research team to reach consensus regarding code definitions, application, and discrepancies in coding, as well as to discuss primary themes as they emerged. In the final phase, the research team organized the coded data based on the NIMHD framework and identified key themes observed across transcripts.

## Results

Table 2 summarizes the qualitative findings organized by NIMHD levels of influence and illustrative quotations. Below, we describe thematic constructs identified including preparation, partnership, prioritization, and placement across levels of influence.

### Preparation

When asked about their knowledge of lung cancer or lung cancer screening, participants expressed uncertainty regarding the screening process and availability in their communities, and shared concerns about the costs associated with screening. Questions were raised about insurance coverage and whether screenings are accessible for those without insurance. Participants also discussed a lack of knowledge regarding the causes of lung cancer (heritability, environmental causes, etc.) and how it is treated.

Participants highlighted a lack of education in their communities about lung cancer and screening, exacerbated by limited communication channels such as billboards, television, and the internet. Participants consistently underscored the significance of education and ensuring information regarding lung cancer risk and screening is readily available, accessible, and relevant. Emphasis was placed on the importance of targeted, local outreach efforts, frequent and widespread messaging, engagement of community members in educational initiatives, and innovative approaches to effectively communicate with individuals within the Black community who may be at higher risk for lung cancer. Various forms of outreach were proposed, such as canvassing, media advertisements, health fairs and mobile outreach, and offering incentives for engagement. Participants emphasized the significance of community sharing of information and suggested locations for outreach efforts including schools, churches, gyms, senior centers, and healthcare facilities, underscoring the importance of promoting awareness about lung cancer and screenings within the Black community.

### Partnership

Participants conveyed perceptions of discrimination and mistrust toward healthcare professionals and researchers, especially from different racial or ethnic backgrounds. This mistrust extended to the medical system and the broader government, stemming from historical instances of systemic discrimination and exploitation. This lack of trust poses practical barriers, such as doubts about receiving adequate support after a cancer diagnosis. Concerns were expressed about a perceived lack of urgency, attention, or support from health care providers. Participants reported instances of dismissiveness or insufficient support when requesting lung cancer screening from their clinicians.

For some, stigma presents an obstacle to lung cancer screening. Intersectional stigmatizing factors such as gender, race, socioeconomic status, and smoking history were identified. Participants expressed how stigmatization of smoking may lead to avoidance of screening, especially among Black elders.

Participants highlighted the importance of social support in facilitating access to lung cancer screenings, recommending either a buddy system where individuals attend screenings with a trusted companion, or peer navigation to help people understand the testing process, navigate procedures, and interpret lab results. Participants recommended encouraging social support within families to support individuals who may feel fearful or apprehensive about undergoing screening.

### Prioritization

Participants frequently associated “lung cancer” with death, evoking fears about mortality. This was often informed by family history of illness, such as witnessing family members facing cancer. The potential to receive a cancer diagnosis evoked fear and presented a barrier to pursuing screening, revealing uncertainty about how to manage the potential strain of a cancer diagnosis.

Community beliefs, norms, and perceptions of healthcare were cited as influencing engagement with screening. Participants described a complex relationship with smoking in the Black community, noting that observing others smoking in the community often influences individuals to begin smoking themselves, and that smoking frequently involves social gatherings among community members. Participants also observed that community members can be dismissive, disregarding how smoking affects air quality and its impact on others, and, particularly among young adults, a lack of concern about lung health overall.

Participants described a tendency towards delayed healthcare-seeking behavior, with individuals often seeking care only when symptoms reach a critical stage; a pattern greatly influenced by social determinants of health. Healthcare, especially preventive care, is often deprioritized due to more immediate concerns such as gun violence, economic instability, including challenges with rent and food affordability, and other health priorities.

Despite these fears, worries, and influential beliefs and community norms, participants generally agreed on the importance of lung cancer screening, emphasizing that it is equally important as other health awareness initiatives such as HIV/AIDS and Hepatitis C, and that all forms of cancer screening (i.e., breast, colorectal) hold equal significance. Participants emphasized the importance of increasing awareness about lung cancer and screenings within the Black community. Participants perceived higher risk of lung cancer and elevated tobacco-related illness among Black individuals compared to other racial and ethnic groups and raised concerns about exposure to pollutants stemming from poor air quality, environmental contaminants, and secondhand smoke. Participants reflected a collective belief in the importance of heightened personal health awareness, noting that education and knowledge are essential in empowering individuals to manage their health effectively and potentially save lives. Factors identified as influencing lung cancer screening included familial encouragement, family health history considerations, and observing illness effects and outcomes of inadequate prevention or care via the media or lived experience.

In addition to individual and community-level factors impacting prioritization of lung cancer screening, participants shared perspectives that lung cancer screening is viewed as a lower priority within their healthcare system. Perceptions of greater emphasis on smoking cessation over screening, and more attention given to screening for cancers like breast or colorectal, were commonly cited. Participants reported that healthcare providers rarely initiated discussions about screening during appointments, contributing to the perception that it is not prioritized. Integrating discussions about lung cancer screening into routine check-ups with primary care physicians and following consistent monitoring schedules were proposed as potential solutions. Participants likened this approach to existing practices for other cancer screenings, such as those for colorectal or breast cancer, with doctors initiating conversations and reminders for screening. Another suggestion was to adopt a similar screening approach as seen with other health concerns like COVID-19 and HIV, ensuring accessibility to lung cancer screening comparable to these tests, and increasing outreach and awareness within communities.

### Placement

Participants highlighted that their community experience disparities in information about and access to local lung cancer screening services compared to more affluent neighborhoods. One participant noted that they never see literature or outreach on this topic, and that lung screening services are not advertised. Another shared a perception that there is likely more outreach for screening in upscale areas and noted a lack of care and concern for low-income communities. Participants stressed the importance of locating screening services within local communities for maximum accessibility and convenience, noting that many community members do not frequently travel outside their neighborhoods. .

## Discussion

The study objective was to explore multi-level barriers to, and facilitators of, lung cancer screening among screening-eligible Black community members in NYC. Guided by the NIMHD Research Framework [18], we identified key constructs: preparation, partnership, prioritization, and placement that intersect across individual, interpersonal, community, and societal levels. These findings help contextualize structural inequities and inform strategies for promoting equitable lung cancer screening.

### Preparation

Participants frequently described a lack of awareness about lung cancer screening guidelines, eligibility, and availability, findings that echo prior research demonstrating low screening-related health literacy among Black adults [26]. Such informational gaps likely reflect broader systemic failures in health education and outreach to minoritized communities. These findings underscore the critical role of community-level interventions in disseminating relevant information about lung cancer risk and screening. Culturally relevant education materials and communication approaches can help address the social determinants of health that shape health behaviors and improve engagement among screening-eligible Black individuals.

Participants recommended using diverse outreach channels, such as flyers, social media, and mobile services to effectively reach Black communities. Incentives were seen as helpful in reducing practical barriers like transportation. Suggested locations for outreach included schools, churches, gyms, and healthcare facilities, reflecting where community members naturally gather. Strategically placing educational materials in these spaces can improve accessibility [27], and messaging should be free of stigma to build trust and engagement.

### Partnership

Trusting relationships with health care providers were central to participants’ decisions about lung cancer screening. Mistrust toward healthcare professionals and researchers, particularly from different racial or ethnic backgrounds, extended beyond individual providers to the broader medical system and government institutions, rooted in historical injustices and lived experiences of discrimination and neglect. For example, a study of 34 lung screening-eligible individuals, half of whom identified as Black, found that mistrust of providers contributed to reduced engagement with preventive care, including screening participation [28].

Patient–provider communication played a crucial role in shaping screening decisions. Our findings align with prior research showing that Black patients often report feeling dismissed or inadequately supported in clinical encounters, and experience disparities in communication, such as shorter visits, less patient-centered dialogue, and greater provider verbal dominance compared to White patients [29–31]. These results underscore the need for empathic, trust-building communication that validates patient concerns and promotes psychological safety. Training in implicit bias, cultural competence, and empathic communication may help strengthen patient–provider relationships. Future research should assess whether such efforts improve engagement and reduce screening barriers.

Stigma also emerged as a prominent barrier, consistent with prior research [32]. Participants described how stigma related to gender, socioeconomic status, and smoking history discouraged screening due to fears of judgment or discrimination. Addressing stigma is particularly critical among Black individuals due to their disproportionate burden of lung cancer [8–10]. Community-based health outreach could be adapted to explicitly address stigma and normalize screening discussions [30]. Studies could explore innovative approaches to destigmatize lung cancer screening through public awareness campaigns that amplify survivor voices and normalize the screening process.

### Prioritization

Competing life demands and social determinants of health, such as housing instability, financial insecurity and caregiving responsibilities often led participants to delay their own health needs, including preventive care. Participants also described a perceived lack of prioritization of lung cancer screening within the healthcare system. This reinforced the perception that lung cancer screening is less important. Uncertainty about screening costs and insurance coverage may further discourage care-seeking.

These findings highlight the need for healthcare systems to elevate lung cancer screening as a key component of preventive care. Proactive provider engagement, including initiating discussions, routine reminders, and integration of screening into primary care can help normalize the practice and improve uptake among screening-eligible Black individuals. At the policy level, expanding insurance coverage and eliminating cost-related barriers are essential to improving access.

Psychological barriers, particularly fear of a cancer diagnosis, shaped how participants prioritized lung cancer screening, often fueled by personal or family experiences and uncertainty about outcomes. This fear led to avoidance and fatalism, highlighting the need for outreach that frames screening as a safe and effective tool for early detection. Culturally tailored education, health literacy promotion, and peer-led efforts can help address fears and improve engagement. One study found that while informational leaflets may increase knowledge about lung cancer screening, they may not be sufficient on their own to overcome fear [33]. This underscores the importance of combining knowledge dissemination with trust-building and emotional support strategies to effectively promote screening uptake. Despite these concerns, participants emphasized the value of lung cancer screening, particularly for Black communities disproportionately affected by tobacco-related illness, environmental exposures, and structural barriers to care.

### Placement

Our findings highlight the role of geographic access on lung cancer screening behaviors. Participants described a lack of nearby screening services and minimal visibility of outreach materials in their neighborhoods, contributing to the perception that screening was inaccessible. Many participants cited barriers to traveling outside their immediate neighborhood, including time, cost, and caregiving responsibilities. These barriers reflect broader patterns of resource inequity in cities like NYC, where historically underserved neighborhoods, often impacted by long-standing disinvestment, face persistent barriers to preventive healthcare access [34]. To address placement-related barriers, public health systems must ensure equitable allocation of screening resources across neighborhoods. Investing in infrastructure within historically underserved communities and prioritizing localized, convenient access to screening services can help bridge the gap between awareness and action, ultimately improving uptake and reducing disparities.

Although our study has important findings, it is not without limitations. While our sample allowed us to reach thematic saturation [22], we recognize that the Black community is diverse, and a larger sample could enable deeper stratification across important participant characteristics such as socioeconomic status, gender, ethnicity, and geographic location.

## Figures and Tables

**Table 1:** Sociodemographic characteristics of study participants

Characteristics	(N = 11)
Age	
Mean	64.4
Race	
Black or African American	11 (100.0%)
Gender	
Female	6 (54.5%)
Male	5 (45.5%)
Ethnicity	
Hispanic or Latino/Latina	2 (18.2%)
Not Hispanic or Latino/Latina	9 (81.8%)
Highest Level of Education	
Some high school	3 (27.3%)
Graduated high school or equivalent	4 (36.4%)
Some college	2 (18.2%)
Bachelor’s degree	1 (9.1%)
Post-graduate degree	1 (9.1%)
Marital status	
Single	7 (63.6%)
Married	3 (27.3%)
Separated	1 (9.1%)
Screening history	
Have been screened	2 (18.2%)
Never been screened	9 (81.8%)

**Table 2:** Qualitative findings organized by NIMHD framework

Thematic construct	Code category	Illustrative quotations	NIMHD level of influence
Preparation	Lack of knowledge	“Knowledge. Knowledge. Knowledge. Knowledge. If you don’t have the knowledge or the information to do better, then how are you going to do better?” - Participant 210 “I feel a lot of people are ignorant of the fact that lung cancer can happen to anyone. Like I said before, a lot of people feel relaxed, simply because they are in good health. And they feel they are not at risk of having lung cancer. I feel that’s because of lack of information.” - Participant 205	Individual
Preparation	Lack of outreach and education about lung cancer and lung cancer screening	“I never see lung cancer screenings or anything about smoking at the community health fairs. I don’t know if it’s just the choices that…or people never really think about it. Because usually, the ones that are giving the health fairs are nonsmokers.” - Participant 207 “I don’t see any billboards up saying, coming in here. There’s lung cancer screening. I don’t see any.” - Participant 210	Societal
Preparation	Community-based strategies for outreach and education and recommendations for where and how to conduct outreach	“Educating the community. Don’t come with a bunch of big, old fancy words and stuff, medical terminology. Come like we were sitting at the kitchen table.” - Participant 106 “Well, in the Black community in order to educate people, you’ve got to really be… resourceful, to get all the information to the people that want to stop and information to people that don’t want to stop. Or give them some kind of incentive where they can go and say, hey, let me go do this, so I can find out if I’m positive for cancer or what. And if so, what kind of resource can I get in order to help me to deal with this?” - Participant 209 “When they have a brochure, it’ll give them a breakdown of each stage and whatever the complications of the cancer, how it grows. […] You give them an education on how it grows and stuff like that and how it could be helped. […] How you can get treatment, where you can get the treatment at. […] All that should be on the little brochures they hand out.” - Participant 204 “To make the screening simple enough that a six-year-old could understand it because a lot of things that are presented, it’s too complicated for some people to understand. […] I've learned from my peers to make it as simple as possible for people to understand it because a lot of times, people say they understand but they don’t understand.” - Participant 109 “Everywhere. Everywhere it should be advertised. On the billboards outside, on the television, on the phones, everywhere. On the computers, anywhere and everywhere. In the laundromat where you go was your clothes, wherever. in the subway station, everywhere, everywhere. Buses, trains, cabs, wherever.” - Participant 210	Community
Preparation	Insurance coverage and cost of lung cancer screening	“Some people, if they don’t have insurance, then likely they probably won’t even go [get screened] because they're going to have to pay whatever, you understand what I'm saying? If it’s just an open screening and stuff like that, people will try it out. […] So that would be good for them to have that kind of thing where you could just walk in without insurance and get a screening and help you out.” - Participant 204 “It shouldn’t cost nothing, that’s what I think, if in the Black community, in a ghetto neighborhood, you know what I'm saying? It should be for free. I guess it’s not for free, they've got to charge for it, right?” - Participant 206	Societal
Partnership	Perceptions of discrimination and mistrust among Black community members	“A lot of things happened just like…the African American community went through several different projects that were supposed to be for good that turned out to be, you know-it was horrifying. Like the syphilis epidemic that happened, we were used as experimental guinea pigs where they would give people syphilis. So, a lot of people-just like with this coronavirus, a lot of people don’t want to take it because of previous stuff that the government has issued that was supposed to be prevention, but it was part of destruction.” - Participant 109 “Once people are screening, they find [out] they got any kind of cancer and now they're going to worry more and they're going to think about it. […] They're scared to go to the hospital because some time, the hospital messed up. […] A lot of people don’t trust these doctors these days, that’s what I think.” - Participant 206	Community
Partnership	Stigma	“With our seniors, smoking, they used to hide their smoking because -and depending on what class system they're in, smoking wasn’ tolerated, or smoking was, that was what, as the clients would say, the hoochie mommas do or the girls that were very fly.” - Participant 106 “Some people have been blamed for having smoked in their lifetime, and this might have led to avoidance of screenings, stigmatization…So I feel the blame has been on people who smoke and some of these people have felt bad about it because they felt they were being pushed out or rejected. And this has made them keep to themselves and try to avoid anything about screening.” - Participant 205	Societal
Partnership	Social support for lung cancer screening	“Yeah, a buddy system. You know, let me walk - you're going to do what? You're going to do a screening? Can I come with you, or do you want me to come with you? Or, better yet, could you please come with me? Because I don’t understand all the stuff they're saying. Do you think you understand it?” - Participant 106 “Maybe we can educate some of our families on how to come together in this community to help educate people and talk to them about how to go about getting screened for lung cancer. And then, to mentor some of those who are scared to go get diagnosed or screened for cancer because they're afraid their test might come back positive.” - Participant 209	Individual
Partnership	Clinician-patient communication	I told him, “can you give me a referral for the […] lung screening to get my lungs checked or screened?” And he said […] “you always coming up with something. The last time, it was about your chest and your heart.” Yeah, there’s something going on and you’re my doctor […] I want you to put in a referral, so I can get things checked out. […] So he put in the referral but he put it in for a chest x-ray. And then, I had to go into my notes, into my chart, and tell him that, “you put it in for the wrong thing. I never said I wanted a chest x-ray. Once again, you’re not listening to your patient.” - Participant 108	Interpersonal
Prioritization	Fears and worries	“Then when they get [screened] and they find out they’ve got breast cancer or whatever kind of cancer out of it, I guess they’re going to get scared, you know. I know I would get scared.” - Participant 206 “I’m going to be perfectly honest, in the Black community, people are afraid. Again, like I said, I work with people who are HIV-positive. They don’t want to get tested for HIV because of the fear and the stigma. And you know, just like with cancer, nobody wants to know that they have cancer.” - Participant 207 “They're scared. Once they have that cancer screening, that breast or whatever kind of screen it is, right, they're scared […] First they find out they got it, turned positive, they got more scared. Now I don’t know what to do.” - Participant 206	Individual
Prioritization	Beliefs about the value of lung cancer screening	“You can find out ahead of time what your health status is. So basically, you’re saving your own life.” - Participant 106 “Besides its saving lives, it gives a person the opportunity to be educated on what is going on with them healthwise. A lot of people just don’t think about their lungs. They just go ahead and automatically breathe, which is what we do as humans. But you need to know what’s going on, and a screening is very important.” - Participant 106	Individual
Prioritization	Complex relationship with smoking in the Black community	"They enjoy smoking. We are relationship people… You see people gathered around outside, smoking after a drink… Even though smoking isn’t allowed in bars anymore, people still go outside to smoke and return with cigarette smoke on them, passing it on to others, some of whom may never have smoked.” - Participant 207 “I think it’s challenging because if kids see that their parents smoke, then they feel like it’s alright to smoke. And just like with the herb, if kids see that their parents are smoking herb, it doesn’t matter if you tell the kids not to smoke. They see that you smoke, and they feel that it’s alright for them to do it.” - Participant 109	Community
Prioritization	Perceived lack of concern about lung health in the Black community	“And it’s dangerous now because sometimes you can’t talk to some of these kids to tell them what’s right and what’s wrong because they're going to do what they want to do anyway.” - Participant 109 “I've heard people say, “I'm just not stopping, I don’t want to stop smoking.” Which just sounds ignorant and just dumb to me to say something-even if you felt that way, I wouldn’t say it. But I've heard people say it. […] Some people just don’t want to stop smoking. Some people think it’s sexy and it’s cute.” - Participant 210	Community
Prioritization	Healthcare seeking behavior in the Black community	“Here I'm dealing with lung cancer but let me ask you a question, does the landlord accept the rent check? […] Those are the major concerns. First, is my rent paid? Number two, do I have something to eat? Okay, fine, we have all of that? Okay, let me see what I can do about this lung cancer.” - Participant 106 “My roommate has an ex-girlfriend who has just been told that she has spots on her lungs and all that kind of stuff […] And I said, “Oh my God, how can that happen so fast?” And she said, “I don’t know, it’s like…” She says she was in pain and stuff but she just kind of ignored it […] And that’s really bad, and that’s what we have a tendency to do. We have a tendency to put stuff off because we're taking care of everybody and their momma. And then when it’s time to take care of us, sometimes it’s too late, so that’s it.” - Participant 106	Community
Prioritization	Perceived importance of lung cancer screening due to high illness rates among Black individuals	“We need to be aware, people of color, in regards to the different forms of cancers that we’re dealing with. And I think we've always dealt with them. I think it’s because of social media, we’re now seeing the statistics are higher than we thought or never thought about. So, the screenings are necessary.” - Participant 106 “Now like I said about the Black culture, African American is that we are mostly on the highest rate of that cancer thing… so you could have it in areas, too, where the lower class, middle class, everybody will be…with this thing. Because it’s a very big thing, this lung cancer stuff.” - Participant 204	Community
Prioritization	Concerns about air quality and community exposure	“It doesn’t have to be smokers; it could also be people that was in situations like 9/11 where the air quality is not good, and things like that, and with the train - the rail […] and stuff like that. I think it’s very very, very important that we know the truth and that this screening is available for everyone.” - Participant 208 “Sometimes I see these women walking with their babies and smoking a cigarette.” - Participant 106	Community
Prioritization	Personal motivations for lung cancer screening	“It just doesn’t seem like it could hurt because when me and my wife spoke about this, she was very […] adamant about me doing this […] if you love someone, you make an effort to know your status and be around as long as you can.” - Participant 208 “I had a few people pass away and walking around here all healthy because they only went to the hospital for regular things or regular checkups and wind up dying from full-blown cancer. And it kind of hurt me. Like, they might have knew it later on in life and they couldn’t make no turnaround in it because it was already full-blown… but I don’t want any of them things to happen now that I can get a chance to change something, I want to be able to have a chance to do it.” - Participant 108	Individual
Prioritization	Perception of low priority of lung cancer screening in healthcare	“Always they’re like, “Would you like to quit smoking? You can take a patch, or you can take a pill.” But they don’t ask you would you like to be checked for lung cancer. They don’t say, “Oh well, you are a candidate for being screened for your lungs to make sure you don’t have cancer.” There’s just no information at all.” - Participant 107 “We kind of maybe have an idea if you got to the hospital and you’re constantly coughing, coughing and the doctor say, oh, let me check your lungs. Of course, he’s going to check your lungs if you are always coughing when you’re in his presence. So, he want to put in a referral for that because he see you coughing all the time and a little bit out of breath or something, so they want to check your lungs. But if you’re not doing all of that and you ask to get your lungs checked, you should be able to have it done.” - Participant 108	Societal
Prioritization	Need for lung cancer screening to be more routine and receive greater focus in healthcare	“They know in your records when is the next time for your colonoscopy. They know the next time in your records when it’s mammogram time. They know all these things in your record when it’s time for you to have these things. Why they don’t have a time and a record that you can get lung cancer screening? It should be part of that…list of things that you have to do every year.” - Participant 108 “Doctors should be telling patients that you need to do a screening. Like colon cancer and other cancer.” - Participant 206	Societal
Placement	Lack of access to lung cancer screening services	“Ain’t no use to beating around the bush. I’m implying the ghetto. I’m sure if you go in Manhattan right now on 14th Street or downtown in mid-Manhattan or in […] the more upscale areas of Manhattan, you will see things and billboards and numbers where you can call and get cancer screening. But in the ghetto, they don’t care about people. I don’t feel, that’s my opinion, I don’t feel that they care about the people that’s in the low- income areas.” - Participant 210 “We don’t have enough experience in lung cancer. We don’t have.no flyers, no tables up there, and there’s no kind of literature you can see in the community about the testing for your lungs. There’s just nothing out there really.” - Participant 107	Community
Placement	Lung cancer screening services need to be local to communities	“Traveling to attend the screening and the treatments, I think those are the major barriers.” - Participant 205 “So, a lot of African Americans don’t like to travel really far. So, if you had somebody outside and they was in the Bronx. And then you're telling them, well, you can go over to this walk-in place in Brooklyn, they're not going to go. They're not going to want to go all the way to Brooklyn, jump on the train, just to go get a screening…If they're given the information to a place that’s near where they live at or what area they're at, then that would make it easier for them to walk in.” - Participant 204	Community

## Data Availability

De-identified interview transcripts can be obtained from the corresponding author (W.A.) upon reasonable request, subject to approval by the Institutional Review Boards of Memorial Sloan Kettering and the City University of New York, for the purpose of scholarly inquiry.
